# A time series analysis of detection and mortality of hepatitis C in Brazil, 2008–2018

**DOI:** 10.1186/s12879-022-07063-5

**Published:** 2022-01-24

**Authors:** Rodrigo José Videres Cordeiro de Brito, Leonardo Feitosa da Silva, Márcio Bezerra Santos, Patrícia Muniz Mendes Freire de Moura, Carlos Dornels Freire de Souza, Rodrigo Feliciano do Carmo

**Affiliations:** 1 Postgraduate Program in Health Sciences, Universidade de Pernambuco (UPE), Pernambuco, Brazil; 2 College of Medicine, Universidade Federal Do Vale Do São Francisco (UNIVASF), Pernambuco, Brazil; 3 Department of Medicine, Universidade Federal de Alagoas (UFAL), Alagoas, Brazil; 4 Department of Morphology, Universidade Federal de Sergipe (UFS), Sergipe, Brazil; 5 College of Pharmaceutical Sciences, Universidade Federal Do Vale Do São Francisco (UNIVASF), Av. José de Sá Maniçoba, s/n, Centro, Petrolina, PE Brazil

**Keywords:** Epidemiology, HCV, Hepatitis C, Incidence, Mortality

## Abstract

**Background:**

The 69th World Health Assembly approved the Global Health Sector Strategy to eliminate hepatitis C virus (HCV) infection by 2030. In Brazil, efforts have been undertaken to achieve this goal; there are, however, great challenges. It is important to understand the disease profile in different regions of the country in order to design strategies to fight the disease nationwide. The objective of this study was to analyse the time trend of the incidence and mortality of hepatitis C in Brazil during the period from 2008 to 2018 according to sociodemographic and clinical characteristics.

**Methods:**

All newly diagnosed cases of hepatitis C reported between 2008 and 2018, in all regions of Brazil, were included. The indicators were obtained from the databases of the Brazilian Ministry of Health. For the time series analysis, a joinpoint regression model was used.

**Results:**

Between 2008 and 2018, 136,759 newly diagnosed cases of hepatitis C were reported considering anti-HCV and HCV RNA positivity, and 271,624 newly diagnosed cases were reported considering one or another positive test. The majority of the records were concentrated in the Southeast (61%) and South (26.2%) Regions. The joinpoint regression model indicated an increasing trend in the detection rate of hepatitis C in Brazil, but there was a decreasing trend in the mortality rate during the period analysed.

**Conclusions:**

Differences were observed in the time trend of hepatitis C and in the sociodemographic and clinical characteristics in different regions of Brazil. These data can provide support to design strategies for the elimination of hepatitis C in Brazil, according to regional particularities.

## Background

Hepatitis C is a severe global public health problem. It is estimated that around 71 million people worldwide (1% of the population) live with the hepatitis C virus (HCV) and that approximately 400,000 people die annually as a result of complications of the disease, mainly due to cirrhosis and hepatocellular carcinoma [1]. Brazil is a continent-sized country with pronounced socio-spatial inequalities. The most recent serological survey, conducted between 2005 and 2009, revealed an overall prevalence of anti-HCV antibodies of 1.38% in the capital cities of the five Brazilian macro-regions and the Federal District. Seropositivity ranged from 0.68% in the Northeast Region to 2.10% in the North Region [2].

The World Health Organization’s (WHO) proposal is to reduce new hepatotropic virus infections and their associated mortality by 90% and 65%, respectively, by 2030 [3]. The Brazilian Ministry of Health, in accordance with the WHO, has outlined a national strategy to achieve this goal. The general objective of the Plan for the Elimination of Hepatitis C in Brazil is to expand access to prevention, diagnosis, and treatment of hepatitis C, involving the three spheres of government (federal, state, and municipal), to reduce new infections and mortality [4].

In Brazil, as in the rest of the world, access to healthcare is expensive, and regional inequalities lead to differences in diagnosis and access to treatments. It is, therefore, of fundamental importance to conduct epidemiological studies that contribute to characterization of the disease in different regions of the country, in order to contribute to the design of strategies for combating and eliminating hepatitis C throughout Brazil. There are no recent studies in Brazil on the trend behavior of the disease in its different regions. For this reason, the objective of this study is to analyse the time trend of the incidence and mortality of hepatitis C in Brazil during the period from 2008 to 2018, according to sociodemographic and clinical characteristics.

## Methods

### Design, population, and period

This is an ecological study involving records of hepatitis C in Brazil notified during the period from 2008 to 2018. The year 2019 was not included, as the data are preliminary, and the year 2020 was not included due to the COVID-19 pandemic that has had an impact on the organization of health services and the diagnosis of new cases of HCV [5].

### Study setting

The setting of this study was Brazil, its macro-regions, and federative units. The country is the fifth largest in the world in land area (8.5 million km^2^). In 2020, Brazil had an estimated population of 211.7 million inhabitants, distributed in five macro-regions (North, Northeast, Southeast, Central-West, and South) and 27 federative units (26 states and the Federal District) [6].

Brazil is characterized by its regional inequalities, with the persistence of poverty-related infectious diseases and a growing prevalence of chronic diseases and external causes [7]. This condition is further aggravated by geographic polarization, as a result of subnational inequalities; for instance, the North and Northeast regions have greater social vulnerability than that observed in the Southeast and South regions: in 2019, the average income of employees in the North and Northeast regions was less than BRL 1,000.00, while in other regions it was above BRL 1,500.00. On the other hand, the aging rate is higher in the South and Southeast (11.8% and 11.7%) when compared to the North region (7.2%) [8].

### Variables and data sources

Hepatitis C is a disease of compulsory notification throughout the Brazilian territory. All reported cases are registered by the municipalities in the National System of Notifiable Diseases (SINAN), which is an official information system of the Brazilian Ministry of Health. In addition, mortality data are recorded in the Mortality Information System (SIM) of the Ministry of Health. In the present study, official public data were collected from the SINAN and SIM made available by the Ministry of Health, through the panel of indicators and basic data on hepatitis in Brazilian municipalities [9]. Data published in the 2020 Viral Hepatitis Epidemiological Bulletin were also considered. The complete methodology used for cleaning the data and creating the indicators can be found elsewhere [10].

Confirmed cases of hepatitis C were considered as follows: from 2008 to 2014, individuals with both reactive serological markers (anti-HCV and HCV RNA), and from 2015 to 2018, individuals who tested positive for either marker (anti-HCV or HCV RNA). The change in the notification criteria was adopted by the Brazilian Ministry of Health with the objective of increasing the detection sensitivity of new cases of hepatitis C in the national territory [4]. Despite the change, the Brazilian Ministry of Health database provides data on both confirmation criteria for the entire study period. Therefore, in the present study we present data for both criteria from 2008 to 2018.

Eleven epidemiological indicators, grouped into three categories, were included in the study, as follows:

- Hepatitis C by spatial units (two indicators):
  1. Number and detection rate per 100,000 inhabitants of individuals who were anti-HCV positive and HCV RNA positive;
  2. Number and detection rate per 100,000 inhabitants of individuals who were anti-HCV positive or HCV RNA positive;

b. Hepatitis C by sociodemographic and clinical characteristics (seven indicators):
  1. Number and detection rate of hepatitis C per 100,000 inhabitants by sex (male and female) and sex ratio;
  2. Number and proportion of confirmed cases of hepatitis C by race/colour (White; Black; Asian; Mixed, Indigenous; unknown);
  3. Number and detection rate per 100,000 inhabitants by age group and year of notification (< 5 years; 5–9 years; 10–14 years; 15–19 years; 20–24 years; 25–29 years; 30–34 years; 35–39 years; 40–44 years; 45–49 years; 50–54 years; 55–59 years; 60 years or more);
  4. Number and percentage of hepatitis C by level of education and year of notification (Illiterate; first to fourth grade incomplete; completed fourth grade; fifth to eighth grade incomplete; completed elementary school; secondary school incomplete; completed secondary school; tertiary school incomplete; completed tertiary school; unknown; not applicable);
  5. Number and proportion of confirmed cases of hepatitis C by likely source/mechanism of infection (Sexual; transfusion; drug use; vertical transmission; work accident; haemodialysis; household; others; unknown/left blank);
  6. Number and proportion of confirmed cases of hepatitis C by association with HIV/AIDS (yes; no; unknown);
  7. Number and proportion of confirmed cases of hepatitis C coinfected with HIV by macro-region (North; Northeast; Southeast; South; Central-West);

iii. Cause-specific mortality due to hepatitis C in Brazil (two indicators):
  1. Number of deaths due to hepatitis C and mortality rate (per 100,000 inhabitants) as underlying cause, by year of occurrence and sex;
  2. Number of deaths due to hepatitis C and mortality rate (per 100,000 inhabitants) as the underlying cause, by place of residence and year of occurrence.

For these two indicators, deaths due to hepatitis C were considered as underlying cause B17.1 (acute hepatitis C) or B18.2 (chronic viral hepatitis C) [10].

### Statistical analyses

Initially, descriptive analysis of the variables was conducted. At this stage, indicators were described as absolute and relative frequencies and measures of central tendency (mean and standard deviation). Scatter plots with smoothed regression lines were generated to evaluate the relationship between the Brazilian and regional rates. For comparison of detection rates before and after the change in the notification process for confirmed cases of hepatitis C, Wilcoxon signed-rank tests were applied. In addition, descriptive exploratory spatial analysis was conducted.

For time series analysis, a joinpoint regression model was used [11]. This model tests whether a line with multiple segments is statistically more appropriate for describing the temporal evolution of a dataset in comparison with a straight line or one with fewer segments. Accordingly, when a joinpoint occurs, the model identifies the year. Moreover, the model makes it possible to calculate the annual percent change (APC) and the average annual percent change (AAPC). The results were interpreted in the following manner: significant positive APCs were considered increasing trends, and significant negative APCs were considered decreasing trends; otherwise, when there was no significance, the trend was considered stationary. For configuration of the model, the following parameters were adopted: minimum of zero and maximum of two joinpoints, model selection by the permutation test (4499 permutations), and error autocorrelation based on the data, and heteroscedasticity errors option (weighted least squares) considering homoscedasticity (homogeneity of variance) [11].

For all analyses, significance level of 5% and confidence interval of 5% (95% CI) were considered. The results of the analysis are displayed in graphs, tables and choropleth maps. JASP (version 0.14.1, copyright 2013–2016, University of Amsterdam, Netherlands) and QGIS (2.14.11 Open Source Geospatial Foundation, Beaverton, OR, USA) were used.

### Ethical aspects

As this study used data from the public domain, evaluation by the Research Ethics Committee was waived.

## Results

### Analysis of the hepatitis C trend by spatial units

**Fig. 1:**
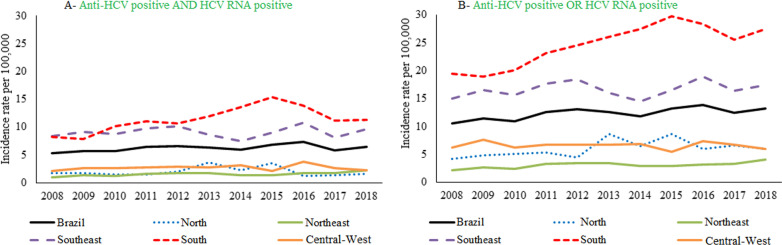
Hepatitis C detection rate considering **A** anti-HCV and HCV-RNA positivity and **B** considering anti-HCV or HCV-RNA positivity, by region of residence and year of notification. Brazil, 2008–2018

**Fig. 2:**
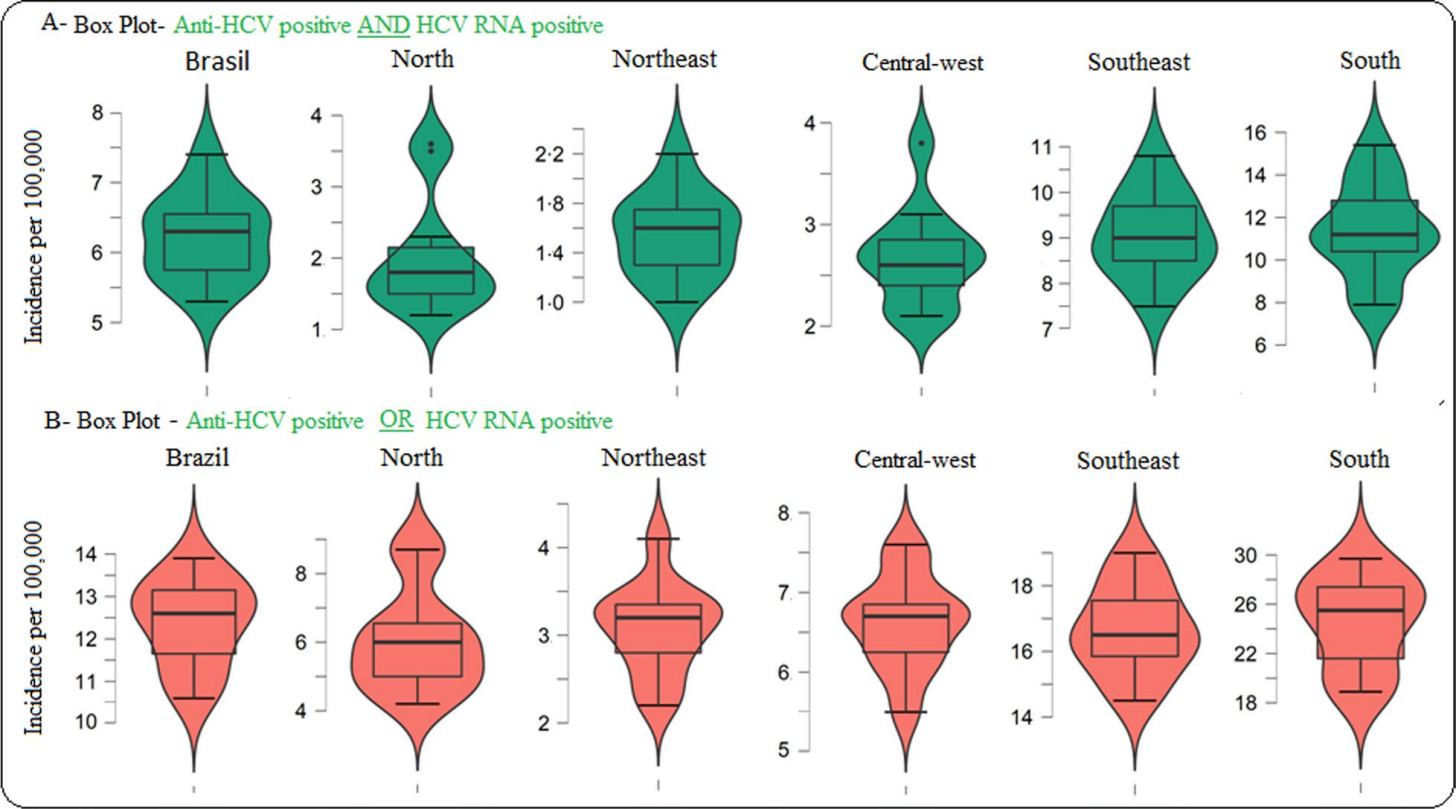
Distribution of hepatitis C detection rate* in the analysed period, considering **A** anti-HCV and HCV-RNA positivity and **B** considering anti-HCV or HCV-RNA positivity. Brazil, 2008–2018. *Each data point represents incidence rate of one year

Between 2008 and 2018, 136,759 newly diagnosed cases of hepatitis C were reported considering anti-HCV and HCV RNA positivity, and 271,624 newly diagnosed cases were reported considering one or another positive test. The records were concentrated in the Southeast Region (61.0%; n = 83,458; 56.4%; n = 153,218, respectively) and the South Region (26.2%; n = 35,870; 28.5%; n = 77,515). The average detection rate in the country was 6.2 ± 0.6 per 100,000 and 12.4 ± 1.0 per 100,000, and it was higher in the South Region, both considering two tests with positive results (11.4 ± 2.3 per 100,000) and considering one or another positive test (24.6 ± 0.60 per 100,000). These two regions remained above the national rate for every year of the time series. On the other hand, the Northeast Region had the lowest average during the period (1.5 ± 0.3 per 100,000 and 3.1 ± 0.5 per 100,000, respectively) (Figs. 1 and 2).

**Fig. 3:**
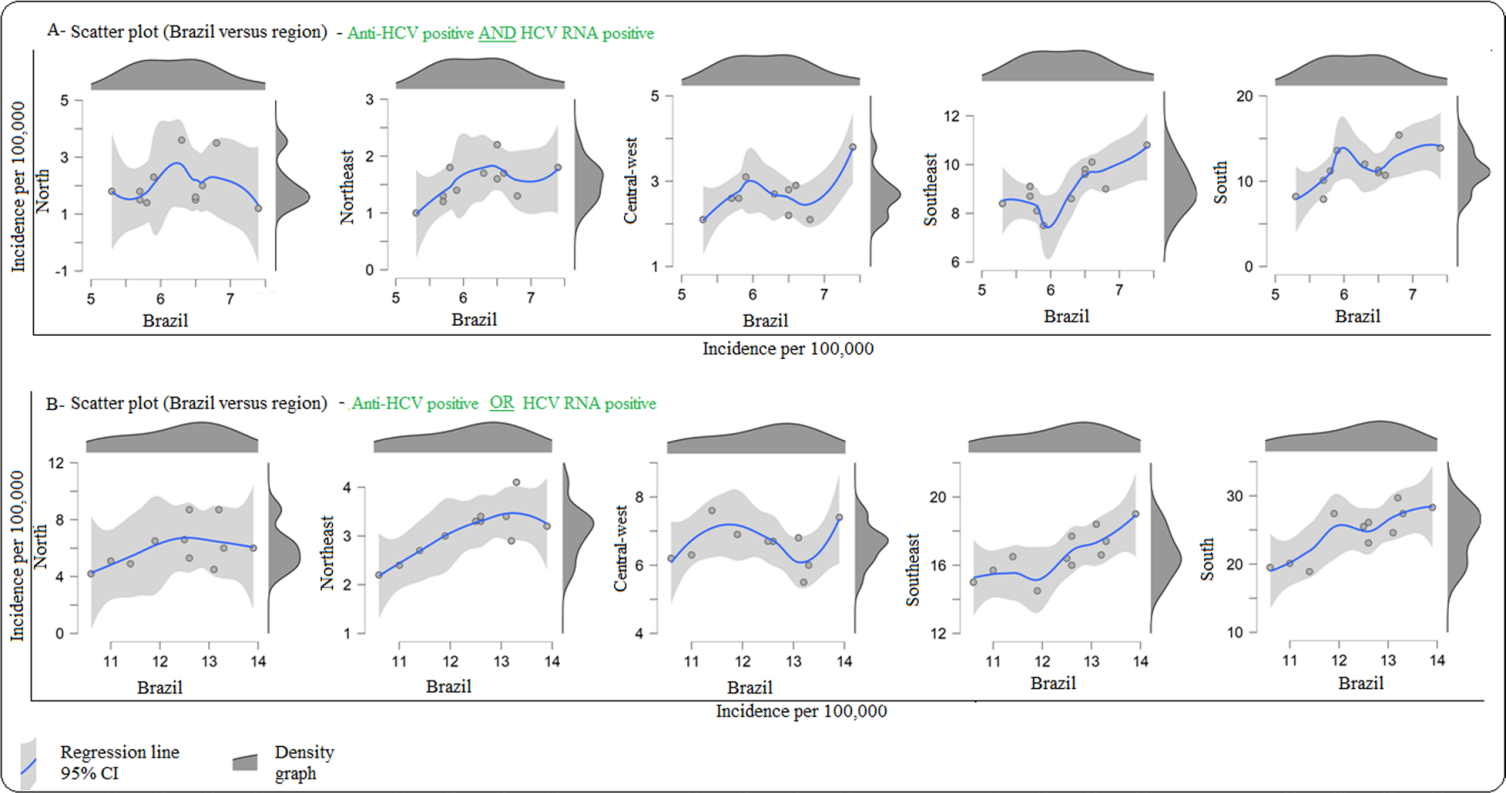
Relation between regional and national hepatitis C detection rate considering **A** anti-HCV and HCV-RNA positivity and **B** considering anti-HCV or HCV-RNA positivity. Brazil, 2008–2018

Furthermore, the density scatter plot displaying the relationship between regional and national rates showed less instability after the modification in the notification process. The Brazilian density curve concentrated values above 13 per 100,000 in the period from 2012 to 2016, reaching 13.9 per 100,000 in the final year of that period. The Central-West Region was the only one with a sinusoidal curve, characterized by growth in the first part of the time series, followed by a decline in the second (Fig. 3).

**Fig. 4:**
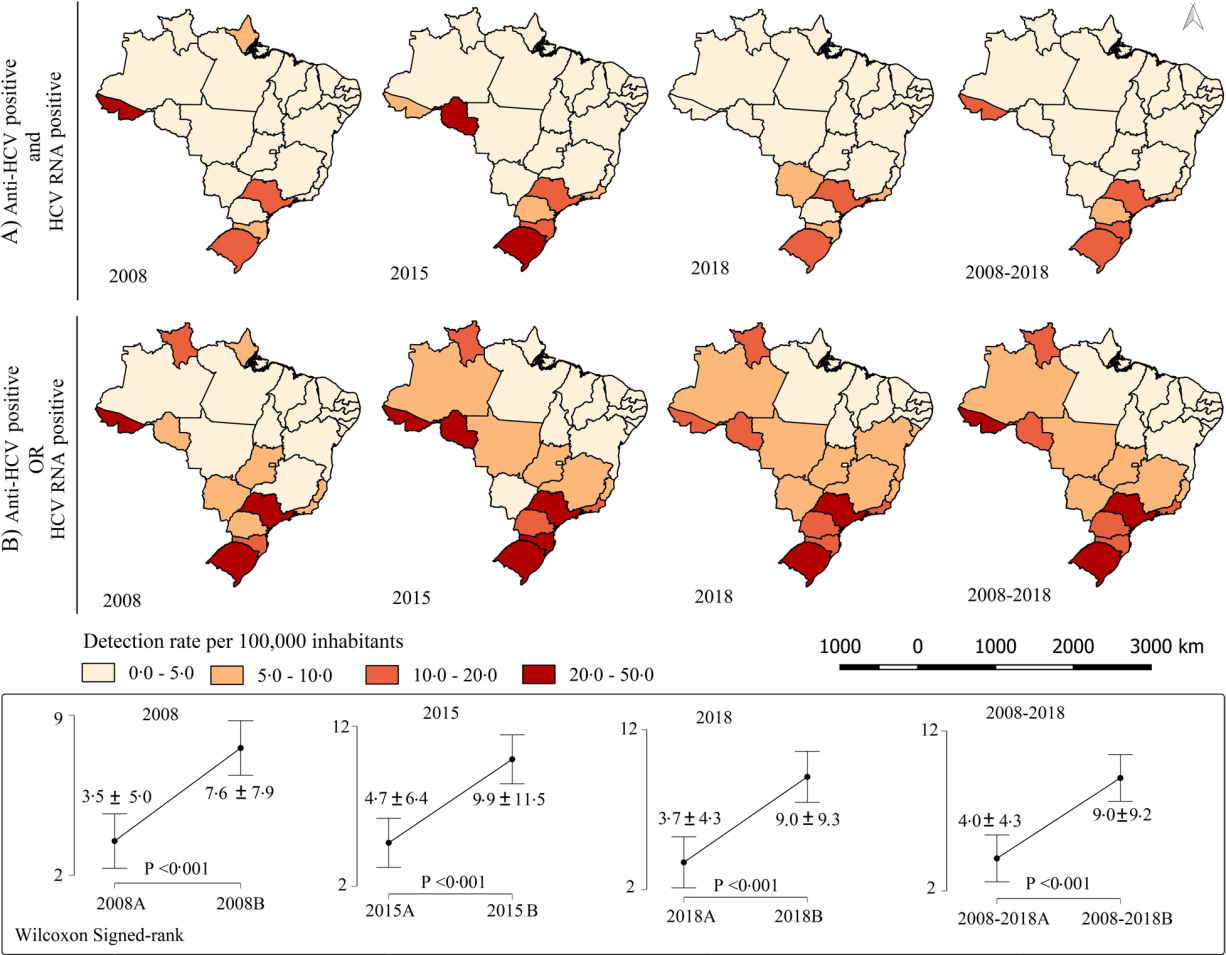
Spatial distribution of the hepatitis C detection rate in Brazil considering **A** anti-HCV and HCV-RNA positivity and **B** considering anti-HCV or HCV-RNA positivity. Brazil, 2008–2018

After the change in the notification process for cases of hepatitis C in Brazil, a significant increase was observed in the states, especially after 2015, the year the change was implemented. In the year 2008, for instance, with the adoption of the criterion of one or another positive marker, the detection rate increased from 3.5 to 7.6 per 100,000, and, in 2018, it went from 3.7 to 9.0 per 100,000. A spatial axis involving the states of the South Region, passing through the Southeast Region and reaching part of the states in the North Region, comprises the geographical units with the highest detection of the disease, especially from 2015 onwards. The highest detection rate was observed in Rio Grande do Sul (17.5 per 100,000 for both positive markers and 40.0 per 100,000 for one or the other), which is far above the national average (Fig. 4).

Considering the complete time series (2008–2018), the joinpoint regression model indicated a higher growth trend in the detection rate of hepatitis C, with one or two serological positive markers in Brazil (APC 2.0%; p < 0.001) and in the North (APC 4.7%; p < 0.001) and South Regions (APC 3.5%; p < 0.001). After the implementation of the change in the mandatory notification process of the disease, a time pattern of growth was observed in ten states: three in the North, starting in 2008 (Rondônia, Amazonas, and Pará); five in the Northeast (Piauí, Ceará, and Bahia, starting in 2008, Alagoas starting in 2013, and Rio Grande do Norte, starting in 2015); one in the Southeast, starting in 2008 (Minas Gerais), and one in the South, also starting in 2008 (Paraná). On the other hand, decreasing trends were observed in four states: three in the North Region from the beginning of the time series (Acre, Roraima, and Amapá) and one in the South starting in 2013 (Santa Catarina) (Table 1).

**Table 1:** Joinpoint regression model for detection rate of hepatitis C considering anti-HCV or HCV RNA positivity and considering anti-HCV and HCV RNA positivity by federative unit and region of residence and year of notification (Brazil, 2008–2018)

Region/federative unit	Anti-HCV positive or HCV RNA positive						Anti-HCV positive and HCV RNA positive					
	Rate		Joinpoint regression model			Trend	Rate		Joinpoint regression model			
	2008	2018	Period	APC/AAPC	95% CI; p value		2008	2018	Period	APC/AAPC	95% CI; p value	Trend
Brazil	10.6	13.3	2008–2018	2.0	0.9 to 3.0; p < 0.001	↑	5.3	6.5	2008–2018	1.6	0.2 to 3.1; p < 0.001	**↑**
North	4.2	6.0	2008–2018	4.7	1.4 to 8.1; p < 0.001	↑	1.8	1.6	2008–2018	− 0.2	− 7.1 to 7.1; p = 0.9	** ↔ **
Rondônia	9.8	11.7	2008–2018	12.2	2.6 to 22.7; p < 0.001	↑	1.9	3.9	2008–2018	17.0	4.4 to 31.0; p < 0.001	**↑**
Acre	24	13.4	2008–2018	− 7.5	− 14.2 to − 0.4; p < 0.001	↓	20.7	0.7	2008–2018	− 26.3	− 35.6 to − 15.6; p < 0.001	↓
									2008–2014	− 8.7	− 22.9 to 8.1; p = 0.2	** ↔ **
									2014–2018	− 46.5	− 61.8 to − 25.1; p < 0.001	↓
Amazonas	2.3	8.1	2008–2018	13.8	6.2 to 21.8; p < 0.001	↑	0.5	3.5	2008–2018	40.5	− 55.3 to 341.8; p = 0.6	** ↔ **
			2008–2013	42.6	26.4 to 60.9; p < 0.001	↑			2008–2013	126.9	− 11.5 to 481.9; p = 0.07	** ↔ **
			2013–2018	− 9.3	− 19.6 to 2.4; p = 0.09	↔			2013–2016	− 62.4	− 99.7 to 5328.4; p = 0.6	** ↔ **
									2016–2018	206.6	− 98.0 to 46,750.5; p = 0.5	** ↔ **
Roraima	11.1	12.6	2008–2018	− 2.7	− 4.8 to − 0.6; p < 0.001	↓	0.7	0.8	2008–2018	−	−	**–**
Pará	1.5	3.4	2008–2018	8.4	3.1 to 13.9; p < 0.001	↑	0.5	0.6	2008–2018	2.1	− 0.6 to 4.9; p = 0.1	** ↔ **
Amapá	8.0	3.8	2008–2018	− 5.1	− 9.5 to − 0.4; p < 0.001	↓	5.7	1.2	2008–2018	− 10.0	− 14.8 to − 4.9; p < 0.001	↓
Tocantins	3.7	2.8	2008–2018	− 0.5	− 3.6 to 2.7; p = 0.7	↔	0.5	0.6	2008–2018	3.3	− 37.5 to 70.7; p = 0.9	** ↔ **
									2008–2010	− 63.9	− 98.7 to 939.0; p = 0.4	** ↔ **
									2010–2014	100.8	− 25.3 to 439.8; p = 0.1	** ↔ **
									2014–2018	− 10.2	− 50.8 to 63.9; p = 0.6	** ↔ **
Northeast	2.2	4.1	2008–2018	5.6	− 2.1 to 13.9; p = 0.2	↔	1.0	2.2	2008–2018	7.8	1.0 to 15.0; p < 0.001	**↑**
			2008–2012	11.8	3.1 to 21.2; p < 0.001	↑			2008–2012	13.5	5.8 to 21.7; p < 0.001	**↑**
			2012–2015	− 6.0	− 34.8 to 35.5; p = 0.6	↔			2012–2015	− 7.6	− 32.5 to 26.5; p = 0.5	** ↔ **
			2015–2018	10.1	− 4.9 to 27.5; p = 0.1	↔			2015–2018	17.4	3.5 to 33.2; p < 0.001	**↑**
Maranhão	2.7	3.1	2008–2018	− 2.7	− 8.6 to 3.6; p = 0.3	↔	1.1	1.3	2008–2018	0.3	− 8.1 to 9.6; p = 0.9	** ↔ **
Piauí	0.2	2.2	2008–2018	28.5	20.3 to 37.3; p < 0.001	↑	0.1	1.5	2008–2018	34.8	24.6 to 45.9; p < 0.001	**↑**
			2008–2011	109.0	64.1 to 166.0; p < 0.001	↑			2008–2011	130.0	71.6 to 208.2; p < 0.001	**↑**
			2011–2018	4.3	− 1.4 to 10.4; p = 0.1	↔			2011–2018	7.2	0.7 to 14.2; p < 0.001	**↑**
Ceará	1.7	3	2008–2018	5.0	2.7 to 7.3; p < 0.001	↑	0.7	1.5	2008–2018	8.3	4.4 to 12.4; p < 0.001	**↑**
Rio Grande do Norte	3.6	3.8	2008–2018	1.9	− 6.1 to 10.6; p = 0.6	↔	2.2	2.1	2008–2018	1.8	− 4.1 to 8.0; p = 0.6	** ↔ **
			2008–2012	− 1.5	− 9.8 to 7.5; p = 0.6	↔			2008–2015	− 7.2	− 11.4 to − 2.8; p < 0.001	↓
			2012–2015	− 12.8	− 41.3 to 29.4; p = 0.3	↔			2015–2018	26.2	1.2 to 57.4; p = 0.04	** ↔ **
			2015–2018	24.8	6.5 to 46.2; p < 0.001	↑						
Paraíba	0.7	3.4	2008–2018	14.3	− 4.0 to 36.1; p = 0.1	↔	0.1	2.4	2008–2018	38.4	12.7 to 69.9; p < 0.001	**↑**
			2008–2010	95.2	− 28.6 to 433.9; p = 0.2	↔			2008–2010	239.8	− 0.6 to 1061.8; p = 0.05	** ↔ **
			2010–2018	− 0.0	− 10.0 to 11.1; p = 1.0	↔			2010–2018	10.6	1.2 to 20.8. p < 0.001	**↑**
Pernambuco	2.0	2.3	2008–2018	2.8	− 6.4 to 12.9; p = 0.5	↔	0.3	0.4	2008–2018	14.2	− 16.3 to 55.9; p = 0.4	** ↔ **
									2008–2012	101.5	− 6.1 to 332.2; p = 0.06	** ↔ **
									2012–2018	− 21.8	− 47.6 to 16.7; p = 0.2	** ↔ **
Alagoas	2	4.2	2008–2018	4.8	− 2.1 to 12.2; p = 0.2	↔	1	1.6	2008–2018	0.9	− 5.3 to 7.6; p = 0.7	** ↔ **
			2008–2013	− 10.3	− 20.5 to 1.1; p = 0.06	↔						
			2013–2108	22.5	8.7 to 38.1; p < 0.001	↑						
Sergipe	3.6	5.1	2008–2018	0.2	− 3.4 to 3.9; p = 0.9	↔	2	3.6	2008–2018	2.3	− 2.2 to 7.0; p = 0.3	** ↔ **
Bahia	2.9	6.9	2008–2018	5.6	4.0 to 7.2; p < 0.001	↑	1.7	4	2008–2018	3.9	0.0 to 7.9; p = 0.04	** ↔ **
Southeast	15	17.4	2008–2018	0.8	− 0.8 to 2.4; p = 0.3	↔	8.4	9.6	2008–2018	0.2	− 1.7 to 2.2; p = 0.8	** ↔ **
Minas Gerais	3.9	8.6	2008–2018	8.6	5.3 to 12.0; p < 0.001	↑	1.6	4.1	2008–2018	7.7	3.3 to 12.2; p < 0.001	**↑**
Espírito Santo	6.2	6.3	2008–2018	3.3	− 0.7 to 7.4; p = 0.09	↔	2.4	2.4	2008–2018	3.4	− 1.6 to 8.7; p = 0.2	** ↔ **
Rio de Janeiro	8	10.9	2008–2018	3.2	− 4.1 to 11.1; p = 0.4	↔	2.7	7.2	2008–2018	9.3	2.1 to 17.0; p < 0.001	**↑**
			2008–2011	22.2	− 5.9 to 58.6; p = 0.1	↔						
			2011–2018	− 4.0	− 10.4 to 2.8; p = 0.2	↔			2008–2011	47.4	15.6 to 87.8; p < 0.001	**↑**
						↔			2011–2018	− 3.8	− 9.6 to 2.4; p = 0.2	** ↔ **
São Paulo	23.9	24.8	2008–2018	− 0.4	− 1.9 to 1.0; p = 0.5	↔	14.3	13.8	2008–2018	− 1.3	− 3.4 to 0.8; p = 0.2	** ↔ **
South	19.5	27.4	2008–2018	3.5	1.3 to 5.7; p < 0.001	↑	8.2	11.3	2008–2018	3.2	0.3 to 6.2; p < 0.001	**↑**
			2008–2015	6.9	4.9 to 8.9; p < 0.001	↑			2008–2018	9.6	7.1 to 12.2; p < 0.001	**↑**
			2015–2018	− 4.0	− 11.1 to 3.7 p = 0.2	↔			2008–2018	− 10.4	− 19.4 to − 0.4; p < 0.001	↓
Paraná	7.8	12.9	2008–2018	6.5	1.6 to 11.7; p < 0.001	↑	2.9	5	2008–2018	7.7	− 0.6 to 16.7; p = 0.06	** ↔ **
			2008–2011	23.9	4.5 to 46.9; p < 0.001	↑			2008–2011	42.1	6.4 to 89.8; p < 0.001	**↑**
			2011–2018	− 0.2	− 4.4 to 4.2; p = 0.9	↔			2011–2018	− 4.3	− 10.9 to 2.7; p = 0.2	** ↔ **
Santa Catarina	16.3	18.4	2008–2018	1.2	− 0.1 to 2.5; p = 0.07	↔	10	8.7	2008–2018	− 0.2	− 3.0 to 2.6; p = 0.9	** ↔ **
			2008–2013	5.6	3.2 to 8.0; p < 0.001	↑			2008–2011	7.0	− 3.6 to 18.7; p = 0.2	** ↔ **
			2013–2018	− 3.0	− 5.2 to − 0.8; p < 0.001	↓			2011–2018	− 3.2	− 5.5 to − 0.8; p < 0.001	↓
Rio Grande do Sul	32.8	47.4	2008–2018	3.2	− 1.1 to 7.7; p = 0.1	↔	12.3	19.2	2008–2018	3.9	− 4.4 to 12.8; p = 0.4	**↔ **
			2008–2010	− 4.0	− 27.4 to 27.0; p = 0.7	↔			2008–2018	5.6	− 3.9 to 16.0; p = 0.2	**↔ **
			2010–2014	12.7	3.3 to 22.9; < 0.001	↑			2008–2018	22.5	− 17.7 to 82.5; p = 0.2	**↔ **
			2014–2018	− 1.9	− 7.0 to 3.4; p = 0.3	↔			2008–2018	− 13.9	− 26.9 to 1.5; p = 0.6	**↔ **
Central-West	6.2	6	2008–2018	− 0.6	− 1.7 to 0.7; p = 0.3	↔	2.1	2.2	2008–2018	0.9	− 1.6 to 3.3; p = 0.4	**↔ **
Mato Grosso do Sul	8.6	9.5	2008–2018	2.3	− 11.3 to 17.9; p = 0.8	↔	1.8	5.2	2008–2018	13.4	− 11.9 to 46.1; p = 0.3	**↔ **
			2008–2013	1.1	− 7.5 to 10.4; p = 0.7	↔			2008–2013	18.5	1.0 to 39.0; p < 0.001	**↑**
			2013–2016	− 29.6	− 61.1 to 27.4; p = 0.2	↔			2013–2016	− 28.6	− 75.3 to 106.9; p = 0.4	**↔ **
			2016–2018	84.7	− 8.2 to 272.0; p = 0.06	↔			2016–2018	103.5	− 40.7 to 597.7; p = 0.2	**↔ **
Mato Grosso	4.6	6	2008–2018	3.4	− 0.1 to 7.0; p = 0.05	↔			2011–2014	6.3	0.5 to 12.4; p < 0.001	**↑**
			2008–2013	13.4	6.6 to 20.5; p < 0.001	↑			2014–2018	41.2	22.8 to 62.4; p < 0.001	**↑**
			2013–2018	− 5.7	− 11.3 to 0.3; p = 0.06	↔			2008–2018	− 12.1	− 18.1 to − 5.6; p < 0.001	↓
Goiás	5.9	5.3	2008–2018	1.4	− 1.4 to 4.2; p = 0.3	↔	2.2	1.5	2008–2018	− 1.6	− 11.3 to 9.2; p = 0.8	**↔ **
									2008–2011	− 10.0	− 26.7 to 10.4; p = 0.2	**↔ **
									2011–2014	18.5	− 28.3 to 95.8; p = 0.4	**↔ **
									2014–2018	− 8.4	− 18.5 to 2.8; p = 0.9	**↔ **
Federal District	6.6	4.3	2008–2018	− 5.1	− 9.8 to 0.1; p = 0.05	↔	3.4	1.4	2008–2018	− 8.4	17.7 to 2.0; p = 0.09	**↔ **

^*^Two years with values of zero, making analysis impossible. Trend classification: ↔ stationary; ↑ increasing; ↓ decreasing. AAPC = average annual percent change. APC = annual percent change. CI = confidence interval

### Analysis of the trend of hepatitis C by sociodemographic and clinical characteristics

In males, the detection rate of hepatitis C increased from 6.4 per 100,000 to 14.9 per 100,000, expressing an increasing trend of 10.8% per year (p < 0.001). In females, the observed rates were lower than in males, but the increasing trend was greater (APC 12.5; p < 0.001), which entailed a reduction in the male-to-female ratio (APC − 1.2; p < 0.001) (Table 2A).

**Table 2:** Detection rate of hepatitis C per 100,000 inhabitants by sociodemographic variables

A) Hepatitis C by sex and sex ratio (detection rate per 100,000 inhabitants)						
Sex	per 100,000		Joinpoint regression model			
	2008	2018	Period	APC/AAPC	95% CI; p value	Trend
Male	6.4	14.9	2008–2018	10.8	6.4 to 15.3; p < 0.001	↑
Female	4.3	11.7	2008–2018	12.5	8.2 to 17.0; p < 0.001	↑
Male-to-female ratio	1.4	1.2	2008–2018	− 1.2	− 1.7 to − 0.7; p < 0.001	↓
Both sexes	5.3	13.3	2008–2018	11.5	7.2 to 16.1; p < 0.001	↑

B) Hepatitis C by age group (detection rate per 100,000 inhabitants)						
Age	per 100,000		Joinpoint regression model			
	2008	2018	Period	APC/AAPC	95% CI; p value	Trend
< 5 years	0.3	1.3	2008–2018	18.5	12.0 to 25.3; p < 0.001	↑
5–9 years	0.1	0.1	2008–2018	−	−	–
10–14 years	0.1	0.4	2008–2018	7.9	− 19.1 to 44.0; p = 0.6	↔
			2008–2013	− 10.1	− 25.1 to 7.9; p = 0.2	↔
			2013–2016	73.2	− 48.5 to 482.6; p = 0.2	↔
			2016–2018	− 16.1	− 79.3 to 240.4; p = 0.7	↔
15–19 years	0.3	2.0	2008–2018	18.3	1.3 to 38.2; p < 0.001	↑
			2008–2013	− 0.2	− 10.3 to 11.0; p = 1.0	↔
			2013–2016	78.7	− 8.2 to 248.1; p = 0.07	↔
			2016–2018	− 2.5	− 52.6 to 100.6; p = 0.9	↔
20–24 years	1.1	4.3	2008–2018	17.7	3.4 to 33.9; p < 0.001	↑
			2008–2013	− 4.0	− 23.6 to 20.7; p = 0.7	↔
			2013–2018	44.1	14.7 to 81.2; p < 0.001	↑
25–29 years	3.0	6.5	2008–2018	9.8	− 0.4 to 21.1; p = 0.6	↔
			2008–2013	− 6.1	− 21.0 to 11.6; p = 0.4	↔
			2013–2018	28.4	8.0 to 52.6; p < 0.001	↑
30–34 years	5.7	9.3	2008–2018	6.2	1.8 to 10.8; p < 0.001	↑
35–39 years	8.1	14.4	2008–2018	7.8	3.2 to 12.5; p < 0.001	↑
40–44 years	11.6	18.9	2008–2018	3.8	2.2 to 5.5; p < 0.001	↑
			2008–2013	− 3.8	− 4.7 to − 2.8; p < 0.001	↓
			2013–2016	28.6	20.4 to 37.4; p < 0.001	↑
			2016–2018	− 8.9	− 15.5 to − 1.7; p < 0.001	↓
45–49 years	13.9	24.9	2008–2018	7.2	3.6 to 11.0; p < 0.001	↑
50–54 years	16.0	31.0	2008–2018	9.1	5.3 to 13.0; p < 0.001	↑
55–59 years	14.8	35.6	2008–2018	10.8	7.2 to 14.5; p < 0.001	↑
60 years or more	8.5	27.9	2008–2018	15.0	10.7 to 19.4; p < 0.001	↑

C) Hepatitis C by race/colour (proportion)						
Ethnicity	%		Joinpoint regression model			Trend
	2008	2018	Period	APC/AAPC	95% CI; p value	
White	71.3	57.5	2008–2018	− 2.1	− 2.4 to − 1.9; p < 0.001	↓
Black	7.2	10.1	2008–2018	3.8	1.1 to 6.6; p < 0.001	↑
			2008–2012	5.3	1.9 to 8.8; p < 0.001	↑
			2012–2016	1.3	− 4.0 to 7.0; p = 0.5	↔
			2016–2018	5.8	− 11.0 to 25.7; p = 0.4	↔
Asian	0.9	0.9	2008–2018	1.5	− 0.3 to 3.3; p = 0.1	↔
			2008–2013	− 1.4	− 4.4 to 1.8; p = 0.3	↔
			2013–2018	4.4	1.2 to 7.7; p < 0.001	↑
Mixed	20.4	31.3	2008–2018	4.2	3.6 to 4.8; p < 0.001	↑
Indigenous	0.2	0.3	2008–2018	9.2	2.9 to 15.9; p < 0.001	↑
Unknown	10.4	11.1	2008–2018	− 0.0	− 2.9 to 3.0; p = 1.0	↔

D) Hepatitis C by level of education (proportion)						
Level of education	%		Joinpoint regression model			
	2008	2018	Period	APC/AAPC	95% CI; p value	Trend
Illiterate	1.0	1.4	2008–2018	4.3	2.2 to 6.4; p < 0.001	↑
First to fourth grade, incomplete	7.5	7.6	2008–2018	− 0.0	− 1.0 to 1.0; p = 1.0	↔
Completed fourth grade	5.9	5.1	2008–2018	− 1.8	− 2.9 to − 0.7; p < 0.001	↓
Fifth to eighth grade, incomplete	16.3	12.6	2008–2018	− 2.3	− 3.1 to − 1.5; p < 0.001	↓
			2008–2011	− 5.9	− 8.1 to − 3.6; p < 0.001	↓
			2011–2015	1.0	− 1.2 to 3.2; p = 0.2	↔
			2015–2018	− 3.0	− 5.3 to 0.6; p = 0.02	↔
Completed elementary school	12.1	8.2	2008–2018	− 4.9	− 6.8 to − 2.9; p < 0.001	↓
			2008–2011	− 11.0	− 17.5 to − 4.0; p < 0.001	↓
			2011–2018	− 2.1	− 3.7 to − 0.5; p < 0.001	↓
Secondary school, incomplete	6.0	5.4	2008–2018	− 0.4	− 1.9 to 1.1; p = 0.6	↔
			2008–2011	− 5.0	− 10.2 to 0.6; p = 0.07	↔
			2011–2018	1.6	0.5 to 2.8; p < 0.001	↑
Completed secondary school	16.8	17.5	2008–2018	− 0.0	− 0.8 to 0.7; p = 0.9	↔
Tertiary school, incomplete	3.1	2.5	2008–2018	− 1..9	− 2.5 to − 1.4; p < 0.001	↓
			2008–2010	− 7.9	− 11.4 to − 4.2; p < 0.001	↓
			2010–2014	− 3.0	− 4.2 to − 1.9; p < 0.001	↓
			2014–2018	2.3	1.6 to 3.1; p < 0.001	↑
Completed tertiary school	7.2	6.8	2008–2018	− 0.6	− 2.7 to 1.6; p = 0.6	↔
			2008–2014	− 4.0	− 6.5 to − 1.4; p < 0.001	↓
			2014–2018	4.9	− 0.6 to 10.7; p = 0.07	↔
Unknown	23.4	32.2	2008–2018	4.1	2.3 to 5.9; p < 0.001	↑
			2008–2011	15.0	8.1 to 22.4; p < 0.001	↑
			2011–2018	− 0.3	− 1.7 to 1.2; p = 0.6	↔
Not applicable	0.6	0.7	2008–2018	3.2	1.8 to 4.5; p < 0.001	↑

Trend classification: ↔ stationary; ↑ increasing; ↓ decreasing. AAPC = average annual percent change. *APC* annual percent change, *CI* confidence interval

Confirmed cases of hepatitis C: until 2014, both anti-HCV and HCV-RNA tests reagent; from 2015 onwards, at least one of the tests reagent, anti-HCV or HCV-RNA

The highest detection rates in 2018 were observed in the age groups above 50 years, with the group between 55 and 59 years standing out (35.6 per 100,000). Increasing trends were observed in children under the age of five (18.5%; p < 0.005) and in all age groups 15 years and older, with the highest APC in the age group from 20 to 24 starting in 2013 (APC 44.1%; p < 0.001). It is worth underscoring that the percentage of growth increased from 40 years of age onwards, reaching 15.0% in the elderly (60 years of age and older) (Table 2B).

With respect to ethnicity, although individuals who identified as White represented the highest percentage of individuals with hepatitis C, this group was the only one with a significantly decreasing trend (APC − 2.1; p < 0.001). On the other hand, the highest annual percentage growth occurred in the Indigenous population (APC 9.2%; p < 0.001), even though this group represented the smallest share of infected individuals (0.3% in 2018). Individuals who identified as Black or mixed race represented 41.4% of infections, with an annual growth trend of 3.8% in the Black group and 4.2% in the mixed race group (Table 2C).

Regarding level of education, although people who are illiterate represented a small proportion of those who were infected (1.4% in 2018), this group showed the highest percentage growth (APC 4.3%; p < 0.001). Individuals with incomplete medical education (APC 1.6%; p < 0.001) and those with incomplete tertiary education (APC 2.3%; p < 0.001) also showed an increasing trend starting in 2011 and 2014, respectively. The growth in individuals whose level of educational was not applicable (children who were not yet literate) was also noteworthy (APC 3.2%; p < 0.001) (Table 2D).

Regarding likely source of infection, a growing trend was observed in fields marked unknown or left blank (APC 4.0%; p < 0.001), the proportion of which increased from 48.0 to 65.6%. Decreasing trends were observed in transfusion transmission (APC − 12.1%; p < 0.001), drug use (APC − 7.6; p < 0.001), work accidents (APC − 6.5; p < 0.001), and haemodialysis (APC − 4.3; p < 0.001). Sexual transmission, although it represented the main source of infection (9.0% in 2018), maintained a stationary time pattern (p = 0.3) (Table 3A).

**Table 3:** Detection rate of hepatitis C per 100,000 inhabitants by clinical variables (Brazil, 2008–2018)

A) Likely source/mechanism of infection (proportion)						
Source/mechanism of infection	%		Joinpoint regression model			
	2008	2018	Period	APC/AAPC	95% CI; p value	Trend
Sexual	7.8	9.0	2008–2018	1.0	− 0.8 to 2.9; p = 0.3	↔
Transfusion	14.2	6.7	2008–2018	− 8.3	− 11.0 to − 5.5; p < 0.001	↓
			2008–2013	− 4.3	− 9.3 to 1.0; p = 0.09	↔
			2013–2018	− 12.1	− 16.7 to − 7.3; p < 0.001	↓
Drug use	16.2	7.9	2008–2018	− 7.6	− 9.7 to − 5.4; p < 0.001	↓
Vertical transmission	0.3	0.2	2008–2018	− 2.7	− 5.8 to 0.6; p = 0.09	↔
Work accident	0.5	0.4	2008–2018	− 6.5	− 9.2 to − 3.7; p < 0.001	↓
Haemodialysis	0.6	0.5	2008–2018	− 4.3	− 6.1 to − 2.5; p < 0.001	↓
Household	0.3	0.6	2008–2018	5.5	− 6.8 to 19.3; p = 0.4	↔
			2008–2010	36.9	− 41.4 to 219.7; p = 0.3	↔
			2010–2014	− 7.1	− 25.8 to 16.3; p = 0.4	↔
			2014–2018	5.0	− 8.5 to 20.6; p = 0.3	↔
Others	12	9.1	2008–2018	− 2.7	− 6.4 to 1.2; p = 0.2	↔
			2008–2013	0.3	− 2.1 to 2.8; p = 0.7	↔
			2013–2016	− 10.5	− 23.9 to 5.3; p = 0.1	↔
			2016–2018	2.3	− 15.3 to 23.5; p = 0.7	↔
Unknown/left blank	48.0	65.6	2008–2018	4.0	3.1 to 4.9; p < 0.001	↑

B) Associated disease (HIV/AIDS) (proportion)						
Associated disease (HIV/AIDS)	%		Joinpoint regression model			
	2008	2018	Period	APC/AAPC	95% CI; p value	Trend
Yes	12.0	6.9	2008–2018	− 5.3	− 12.1 to 2.1; p = 0.2	↔
			2008–2013	− 7.4	− 12.5 to − 2.0; p < 0.001	↓
			2013–2016	4.8	− 24.3 to 45.1; p = 0.7	↔
			2016–2018	− 14.0	− 38.5 to 20.3; p = 0.2	↔
No	73.0	79.5	2008–2018	0.8	0.3 to 1.3; p < 0.001	↑
Unknown	15.0	13.7	2008–2018	− 0.7	− 3.6 to 2.3; p = 0.6	↔
			2008–2011	6.4	− 4.3 to 18.3; p = 0.2	↔
			2011–2018	− 3.6	− 6.1 to − 1.0; p < 0.001	↓

C) Proportion of hepatitis C-HIV coinfection							
Hepatitis C-HIV coinfection	%		Joinpoint regression model				
	2008	2018	Period	APC/AAPC	95% CI; p value	Trend	
Brazil	12	6.9	2008–2018	− 5.3	− 12.1 to 2.1; p = 0.2	↔	
			2008–2013	− 7.4	− 12.5 to − 2.0; p < 0.001	↓	
			2013–2016	4.8	− 24.3 to 45.1; p = 0.7	↔	
			2016–2018	− 14.0	− 38.5 to 20.3; p = 0.2	↔	
North	4.1	3.5	2008–2018	4.1	− 0.8 to 9.2; p = 0.09	↔	
Northeast	3.1	4.6	2008–2018	3.7	− 3.4 to 11.4; p = 0.3	↔	
Southeast	12	6.2	2008–2018	− 4.7	− 7.5 to − 1.8; p < 0.001	↓	
South	15.8	9.3	2008–2018	− 4.5	− 5.7 to − 3.3; p < 0.001	↓	
Central-West	8.9	6.0	2008–2018	− 1.8	− 3.8 to 0.3; p = 0.008	↔	

Trend classification: ↔ stationary; ↑ increasing; ↓ decreasing. AAPC = average annual percent change. *APC* annual percent change, *CI* confidence interval

Confirmed cases of hepatitis C: until 2014, both anti-HCV and HCV-RNA tests reagent; from 2015 onwards, at least one of the tests reagent, anti-HCV or HCV-RNA

Three joinpoints were observed in the proportion of HIV/AIDS associated with hepatitis C. In the first, between 2008 and 2013, there was a decline in the proportion of patients who were HIV-positive (APC − 7.4%; p < 0.001), and it continued stationary from then on. It is worth underscoring that this indicator has improved, and the proportion of fields marked unknown decreased starting in 2011 (APC − 3.6; p < 0.001) (Table 3B). Regarding regions, although the South and Southeast showed the highest proportions of coinfections (9.3% and 6.2% in 2018, respectively), they were the only ones with decreasing trends (− 4.5% and − 4.7%, respectively). The other regions maintained a stationary time pattern (Table 3C).

### Analysis of the trend in cause-specific mortality due to hepatitis C in Brazil

During the period from 2008 to 2018, 21,233 deaths were registered due to hepatitis C in Brazil, with an average cause-specific mortality rate of 0.96 per 100,000 inhabitants for the period. The national trend shows a decline, especially starting in 2013, with a greater annual percentage reduction (APC − 13.1%; p < 0.001). Of these deaths, 60.6% (n = 12,780) were male (1.20 per 100,000 inhabitants), with a male-to-female ratio of 1.5. Considering both sexes, mortality showed a decreasing trend between 2008 and 2018 (APC − 2.9%; p < 0.001). In the male population, the time model showed a decline as of 2015 (APC − 11.1; p < 0.001), and, in the female population, the decline began the following year (APC − 13.4; p < 0.001) (Table 4).

**Table 4:** Mortality rate due to hepatitis C (per 100,000 inhabitants) as underlying cause by (A) sex and (B) region and federative unit of residence (Brazil, 2008–2018)

A) Mortality by sex						
Mortality rate	Per 100,000		Joinpoint regression model			
	2008	2018	Period	APC/AAPC	95% CI; p value	Trend
Male-to-female ratio	1.7	1.5	2008–2018	− 0.4	− 1.5 to 0.6; p = 0.4	↔
Male	1.3	0.9	2008–2018	− 3.2	− 4.7 to − 1.5; p < 0.001	↓
			2008–2015	0.5	− 0.9 to 1.9; p = 0.4	↔
			2015–2018	− 11.1	− 16.4 to − 5.6; p < 0.001	↓
Female	0.7	0.6	2008–2018	− 1.1	− 1.1 to − 1.1; p < 0.001	↓
			2008–2010	8.4	8.4 to 8.4; p < 0.001	↑
			2010–2016	0.2	0.2 to 0.2; p < 0.001	↑
			2016–2018	− 13.4	− 13.4 to − 13.4; p < 0.001	↓
Both sexes	1.0	0.8	2008–2018	− 2.9	− 2.9 to − 2.9; p < 0.001	↓
			2008–2016	− 0.2	− 0.2 to − 0.2; p < 0.001	↓
			2016–2018	− 13.1	− 13.1 to − 13.1; p < 0.001	↓

B) Mortality by region and federative unit						
Region/federative unit	Anti-HCV positive or HCV RNA positive					
	Rate		Joinpoint regression model			Trend
	2008	2018	Period	APC/AAPC	95% CI; p value	
Brazil	1.0	0.8	2008–2018	− 2.9	− 2.9 to − 2.9; p < 0.001	↓
			2008–2016	− 0.2	− 0.2 to − 0.2; p < 0.001	↓
			2016–2018	− 13.1	− 13.1 to − 13.1; p < 0.001	↓
North	0.4	0.6	2008–2018	3.4	− 1.7 to 8.7; p = 0.2	↔
			2008–2011	19.4	− 0.8 to 43.7; p = 0.06	↔
			2011–2018	− 2.8	− 6.8 to 1.4; p = 0.2	↔
Rondônia	0.7	1.0	2008–2018	7.5	0.3 to 15.2; p < 0.001	↑
Acre	2.8	3.4	2008–2018	0.2	− 5.4 to 6.0; p = 0.9	↔
Amazonas	0.4	0.6	2008–2018	4.3	− 3.1 to 12.3; p = 0.3	↔
			2008–2011	24.7	− 5.6 to 64.6; p = 0.1	↔
			2011–2018	− 3.3	− 8.5 to 2.1; p = 0.2	↔
Roraima	0.2	0.8	2008–2018	–	–	–
Pará	0.3	0.4	2008–2018	3.9	− 4.0 to 12.6; p = 0.3	↔
			2008–2010	37.1	− 13.8 to 118.5; p = 0.1	↔
			2010–2018	− 3.0	− 7.3 to 1.4; p = 0.1	↔
Amapá	0.5	0.1	2008–2018	− 3.3	− 11.0 to 5.2; p = 0.4	↔
Tocantins	0.1	0.1	2008–2018	–	–	–
Northeast	0.4	0.3	2008–2018	− 1.3	− 3.6 to 1.1; p = 0.3	↔
Maranhão	0.3	0.2	2008–2018	− 4.4	− 18.3 to 12.0; p = 0.6	↔
			2008–2016	5.3	− 1.6 to 12.7; p = 0.1	↔
			2016–2018	− 35.0	− 74.8 to 67.5; p = 0.3	↔
Piauí	0.1	0.2	2008–2018	1.5	− 14.8 to 21.0; p = 0.9	↔
			2008–2015	18.3	2.1 to 37.0; p < 0.001	↑
			2015–2018	− 28.9	− 62.6 to 35.2; p = 0.2	↔
Ceará	0.2	0.2	2008–2018	0.0	− 6.1 to 6.5; p = 1.0	↔
Rio Grande do Norte	0.4	0.3	2008–2018	− 0.8	− 5.1 to 3.7; p = 0.7	↔
Paraíba	0.3	0.5	2008–2018	− 0.4	− 5.5 to 5.1; p = 0.9	↔
Pernambuco	0.7	0.5	2008–2018	− 2.6	− 4.9 to − 0.3; p < 0.001	↓
Alagoas	0.2	0.3	2008–2018	− 2.7	− 8.0 to 2.9; p = 0.3	↔
Sergipe	0.5	0.3	2008–2018	− 3.7	− 31.2 to 34.8; p = 0.8	↔
			2008–2010	− 39.6	92.3 to 375.3; p = 0.6	↔
			2010–2018	8.3	− 1.4 to 18.9; p = 0.08	↔
Bahia	0.4	0.4	2008–2018	1.1	− 1.9 to 4.2; p = 0.4	↔
Southeast	1.4	0.9	2008–2018	− 4.3	− 7.8 to − 0.6; p < 0.001	↓
			2008–2016	− 1.5	− 2.9 to − 0.1; p < 0.001	↓
			2016–2018	− 14.6	− 32.0 to 7.2; p = 0.1	↔
Minas Gerais	0.5	0.4	2008–2018	0.3	− 2.3 to 2.9; p = 0.8	↔
Espírito Santo	0.6	0.5	2008–2018	− 1.5	− 6.7 to 4.0; p = 0.5	↔
Rio de Janeiro	1.9	1.1	2008–2018	− 4.4	− 6.6 to − 2.1; p < 0.001	↓
			2008–2015	− 0.4	− 2.2 to 1.5; p = 0.6	↔
			2015–2018	− 13.2	− 20.5 to − 5.2; p < 0.001	↓
São Paulo	1.7	1.1	2008–2018	− 4.8	− 8.7 to − 0.7; p < 0.001	↓
			2008–2016	− 2.0	− 3.5 to − 0.5; p < 0.001	↓
			2016–2018	− 15.1	− 34.4 to 9.8; p = 0.2	↔
South	1.6	1.3	2008–2018	− 1.4	− 3.1 to 0.3; p = 0.1	↔
			2008–2013	3.0	− 0.1 to 6.2; p = 0.06	↔
			2013–2018	− 5.7	− 8.5 to − 2.7; p < 0.001	↓
Paraná	0.7	0.5	2008–2018	− 1.4	− 4.8 to 2.2; p = 0.4	↔
			2008–2014	8.3	3.8 to 13.1; p = 0.0	↔
			2014–2018	− 14.3	− 21.6 to − 6.3; p < 0.001	↓
Santa Catarina	1.0	0.6	2008–2018	− 5.6	− 7.3 to − 3.9; p < 0.001	↓
Rio Grande do Sul	2.8	2.5	2008–2018	− 0.2	− 1.7 to 1.3; p = 0.8	↔
Central-West	0.5	0.6	2008–2018	− 0.0	− 2.5 to 2.6; p = 1.0	↔
			2008–2013	7.1	2.4 to 12.0; p < 0.001	↑
			2013–2018	− 6.6	− 10.7 to − 2.3; p < 0.001	↓
Mato Grosso do Sul	0.6	1.0	2008–2018	3.1	− 1.3 to 7.8; p = 0.1	↔
Mato Grosso	0.4	0.5	2008–2018	0.1	− 4.9 to 5.3; p = 1.0	↔
Goiás	0.5	0.6	2008–2018	0.8	− 4.5 to 6.3; p = 0.8	↔
			2008–2012	10.9	− 3.1 to 27.1; p = 0.1	↔
			2012–2018	− 5.5	− 11.5 to 0.9; p = 0.08	↔
Federal District	0.8	0.5	2008–2018	− 4.3	− 9.6 to 1.4; p = 0.1	↔

Trend classification: ↔ stationary; ↑ increasing; ↓ decreasing. Deaths due to hepatitis C: underlying cause B17.1 (acute hepatitis C) or B18.2 (chronic viral hepatitis C). *AAPC* average annual percent change, *APC* annual percent change, *CI* confidence interval

Two regions showed a decreasing trend in mortality: the South (APC − 5.7%; p < 0.001) and the Central-West (APC − 6.6%; p < 0.001) starting in 2013. Although the Southeast Region had a decreasing trend (APC − 4.3%; p < 0.001) considering the entire time series (2008–2018), in 2016, it began to present a stationary pattern (p = 0.1). With respect to Brazilian states, Rondônia was the only one with a growing trend (APC 7.5%; p < 0.001). On the other hand, decreasing trends were observed in Pernambuco (APC − 2.6%; p < 0.001) and Santa Catarina (APC − 5.6%; p < 0.001) starting in 2008, in Paraná (APC − 14.3%; p < 0.001) starting in 2014, and in Rio de Janeiro (APC − 13.2%; p < 0.001) starting in 2015 (Table 4).

## Discussion

This study has presented important data regarding the detection and mortality rate of hepatitis C in Brazil in recent years, as well as differences in the epidemiological profile of the disease in different regions of the country.

Mandatory notification of hepatitis C was implemented by the Brazilian surveillance system in 1996. Until 2014, individuals with anti-HCV reactive serology in addition to detectable HCV-RNA were considered confirmed cases. However, since 2015, in order to increase the sensitivity of new case detection, the criteria have been modified, and every individual with at least one of the reagent tests (Anti-HCV or HCV-RNA) is now considered a confirmed case. These changes are in line with the goals of the hepatitis C Elimination Plan in Brazil, which aims to estimate the number of hepatitis C cases nationwide based on epidemiological data, and to expand access to treatment and diagnosis [4]*.* Consequently, starting in 2015, an increase was observed in the detection rate throughout the national territory, with emphasis in the South and Southeast Regions, which remained above the national rate for every year in the time series. Higher detection rates have been reported in these two regions in previous studies [2, 12, 13], and they may be explained by the fact that the regions are populous, and they have greater access to health services [13, 14]. Another factor that may explain the high rates of hepatitis C in the South and Southeast Regions of Brazil is the high rate of people who use injected drugs, given that this factor is strongly associated with positivity for hepatitis C [15, 16].

The HCV-RNA molecular test requires adequate infrastructure and specialized labour; thus, these tests are carried out by state central laboratories located in capital cities. With the more flexible confirmation criteria and the mass distribution of rapid tests to municipal testing centres in recent years [17], there has been greater capacity for detection of new cases in municipalities with less health infrastructure. This has been made evident by the spatial analysis conducted in this study, which indicates an increase in the detection rate along a spatial axis that involves the South, Southeast, Central-West, and North Regions (Fig. 4), in addition to a time pattern of growth in ten Brazilian states. It is important to highlight that the change in the criterion for confirming cases occurred only at the level of epidemiological surveillance. A reactive result to the anti-HCV assay in a sample represents evidence of prior contact with HCV. It is known that approximately 80% of people infected with HCV will become chronic carriers of the infection [18]. For this reason, the Brazilian Ministry of Health has recommended that the results of the anti-HCV assay should be complemented with the use of an assay for direct detection of the viral agent (HCV RNA or HCV-Ag) [19].

In relation to sociodemographic characteristics, a higher incidence of HCV was observed in males. Higher prevalence of HCV in men is related to greater exposure to risk factors, such as the use of inhaled drugs, injected drugs, and sexual activity [20, 21]. In this study, we observed an increasing trend in the detection rate in both sexes; however, there was a decrease in the male-to-female ratio due to a greater increase in the number of cases in women during the period analysed. This trend had already been observed in a previous study carried out in Rio de Janeiro, Brazil, which observed a decrease in the male-to-female ratio, from 1.2 to 0.9, in cases of HCV between the years 2008 and 2012 [21]. The increase in cases among women may reflect behavioural changes over the years, where women may have been more exposed to risk factors for infection. Studies have shown that women who use injected drugs practice behaviours with higher risks of exposure, including higher rates of sharing equipment and syringes [22, 23]. In line with this, a recent meta-analysis of 28 studies showed that women who use injected drugs have a 36% greater risk of HCV infection compared to men [24].

In relation to age, the highest detection rates were observed in individuals over 50 years old, with the age group from 55 to 59 years standing out (35.5 cases per 100,000 in 2018). It is important to observe that there was a significant increasing trend in the detection rate among young adults between 20 and 29 years old, especially between 2013 and 2018. It is also worth highlighting that there was an increasing trend in individuals under 5 years of age and in those over 60 years (APC 18.5 and 15.0, respectively). Previous studies have demonstrated a higher prevalence of HCV in young people and in adults over 45 years old [2, 13, 20, 25]. The main explanation for the increased incidence of HCV in these age groups involves changes in the mechanisms of disease transmission over the years.

Hepatitis C is a silent disease, where most individuals develop the chronic form [18]. Many individuals became infected before 1992, when screening still did not occur in blood banks [26]. The use of glass syringes that were reused for application of drugs was also an important source of contamination before the 90 s [27, 28]. This would explain the increased diagnosis in elderly individuals who were exposed many years ago. In the young population, on the other hand, risk of exposure to HCV is due to the sharing of drug use equipment (syringes, needles, and pipes) and frequent tattooing and piercing without due attention to sterilization or use of disposable equipment [13]. These findings corroborate a trend of change in the source of infection, where we observed a reduced transfusion transmission from 2008 to 2018, and an increase in sexual transmission and by drug use. However, it is important to highlight the fragility of this variable, given that it is difficult to establish the true source of infection. This is reflected in the increase in the proportion of fields marked unknown during the period analysed. In addition, the increase in the detection rate in children under 5 years of age may reflect greater access to prenatal care for pregnant women in recent years. Vertical transmission occurs in up to 5% of newborns whose mothers are infected; this is influenced by the mother’s viral load, by the HIV status, and by the immunogenetic profile of the mother and child [29].

Although the majority of individuals who were infected identified as White, during the period analysed, a decline was observed in the proportion of those who identified as White, in addition to an increase in those who identified as Black, mixed, and Indigenous. This trend of change in the race profile may reflect greater access to hepatitis C diagnosis over the years in the regions in the North of the country, as well as in more vulnerable populations. As demonstrated through spatial analysis, at the beginning of the analysed period, hepatitis C was more concentrated in the South and Southeast Regions of the country, which are the regions with the highest human development indices and those with greater more individuals who identify as White [12, 14]. With increased access to diagnostic tests and more flexible confirmation criteria, the regions in the North of the country, where there are more individuals who identify as mixed race and Indigenous and lower human development indices [14, 30, 31], have led to changes in the disease profile in Brazil. This finding is corroborated by the data on level of education, where an increase was observed in the proportion of individuals who were illiterate and those who had not completed secondary or tertiary education.

About 30% of all people who are HIV-positive have been involved with use of injected drugs, and 85% of these individuals who use drugs are infected with both HIV and HCV [12]. We observed a higher prevalence of HCV-HIV coinfection in the Southeast and South Regions of Brazil, with a decreasing trend in the country. The South had the highest rate, which was greater than the national rate (9.3%). These findings may reflect the high degree of urbanization in the South and Southeast Regions of Brazil, which favours a greater prevalence of people who use injected drugs, which are an important source of hepatitis C and HIV infections [12, 32]. In 2020, Nutini et al. demonstrated a higher prevalence of HCV-HIV coinfection in the South Region of Brazil, where the mean age of coinfected individuals was lower than that of individuals who were monoinfected with HCV [33]. On the other hand, the decreasing trend in HCV-HIV coinfection in the country may reflect increased access to diagnostic tests over the years, as the prevalence of HCV-HIV coinfection could have been overestimated in previous years, due to a greater demand for test orders in individuals who were previously infected with HIV.

During the period from 2008 to 2018, 21,233 deaths were registered due to hepatitis C in Brazil, representing an average mortality of 0.96 per 100,000 inhabitants, with a predominance of males (60.6%). In general, a decreasing trend was observed for mortality nationwide, and it was more significant starting in 2016. Two regions showed a decreasing trend in mortality: the South and the Central-West starting in 2013. This decreasing trend in mortality due to hepatitis C in Brazil may be explained by the introduction of new direct-acting antiviral agents in recent years, which have greater efficacy in treating HCV and have significantly contributed to the goal of eliminating HCV worldwide [20]. These medications were introduced in Brazil in 2013, and, since then, the Brazilian Ministry of Health has updated its clinical protocol and therapeutic guidelines with the inclusion of new medications and expanded access to treatment, which, since 2017, has included all diagnosed cases of chronic hepatitis C in Brazilian territory [34]. It is important to note that the present study only considered those whose underlying cause of death was acute (B 17.1) or chronic (B 18.2) hepatitis C. Therefore, the causes of death recorded as a complication or aggravation associated with HCV, such as liver cirrhosis or liver cancer, may cause an underestimation of the HCV mortality rate found here [35, 36].

On the other hand, as a country, Brazil faces major challenges to reaching the goal of eliminating hepatitis C by 2030, especially in view of challenges posed by the COVID-19 pandemic in 2020, which has affected the control programs for several infectious diseases in Brazil, including HCV [5, 37, 38]. The worldwide impacts of the COVID-19 pandemic have jeopardized all the advances observed in recent years, not only due to the evident impact on the entire health system, but also due to the lack of investigations that evaluate the relationship between the two viruses.

There are some limitations to this study, including the use of secondary data. There is a possibility of underreporting, given that asymptomatic individuals are rarely tested, in addition to the possibility of incorrect records with incomplete data. Regional differences in the quality of surveillance systems directly influence data quality, given that underreporting is greater in more vulnerable areas and in those with less access to health services. In 2010, some states, especially those located in the North and Northeast regions, still had coverage below 90% and proportions of deaths classified as “ill-defined causes” above 10% [39]. Lastly, the notification system does not allow for separate analysis according to the diagnostic method (anti-HCV or HCV-RNA), so we were unable to differentiate between active and past infection in our analyses. This may have influenced, at least in part, the increasing trends observed for some indicators, especially after 2015, when the confirmation criteria was changed in Brazil.

## Conclusion

This study provides important data regarding the behaviour of hepatitis C in Brazil over a 10-year period. A change was observed in the epidemiological profile of the disease, caused mainly by changes in diagnostic confirmation criteria and the introduction of new medications that have contributed to reduced mortality in recent years. Also evident are the differences in the disease profile in different regions of Brazil, as a result of regional differences related to the sociodemographic profile and health and social infrastructure conditions.

An active surveillance system is needed, with strategies developed according to the population profile of each region of Brazil, with expanded diagnosis for all risk groups, in addition to the development of prevention strategies through health education, which will make it possible to slow contagion and promote awareness so that the population will seek testing centres. A joint effort between governments, medical societies, and the industry is also needed to guarantee access to treatment for all individuals who are infected. Only then will it be possible to eliminate hepatitis C in Brazil.

## Data Availability

The following data sources were used in this study: the “Indicators and Data on Hepatitis in Brazilian Municipalities” platform of the Department of Chronic Conditions and Sexually Transmitted Infections of the Brazilian Ministry of Health, available at http://indicadoreshepatitiss.aids.gov.br/,and the epidemiological bulletin titled *“*Viral Hepatitis 2020” of the Brazilian Ministry of Health, a special issue published in July 2020—http://www.aids.gov.br/pt-br/pub/2020/boletim-epidemiologico-hepatites-virais-2020.

## Abbreviations

AAPC: Average annual percent change
AIDS: Acquired immunodeficiency syndrome
ANTI-HCV: Antibodies to the hepatitis C virus
APC: Annual percent change
HCV: Hepatitis C virus
HIV: Human immunodeficiency virus
RNA: Ribonucleic acid
UFAL: University of Alagoas State
UPE: University of Pernambuco State
UFS: University of Sergipe State
UNIVASF: University of São Francisco River Valley
